# Supplementary material for: Dissecting the steps in early simian immunodeficiency virus dissemination following mucosal and intravenous infection of rhesus macaques
Source: J Virol. 2026 Feb 18;100(3):e01485-25. doi: 10.1128/jvi.01485-25 (PMC13011353; doi:10.1128/jvi.01485-25)
Supplement: Supplemental material — Table S1, Fig. S1, and Text S1 to S3. [file jvi.01485-25-s0001.docx]

1. Quantitative summary of experimental setups

| Experimental Setup | Number of animals or replicates | Number of lineages detected (range) | Sequencing time point (day post infection) | Maximum fold-difference in size | Mean variance of log_10_ lineage size ($\sigma_{10}^{2}$)* | Mean variance of log_10_ lineage size prior to removal of tail*^ | Original publication |
| --- | --- | --- | --- | --- | --- | --- | --- |
| Mucosal (intravaginal) inoculation | 5 | 2-8 | 6 (n = 1), 7 (n = 1),  8 (n = 1),  or 14 (n = 2) | 1.09×10^3^ | 1.24 ± 0.54 |  | (1) |
| Intravenous (100 IU) inoculation | 2 | 4-17 | 8 | 1.73×10^3^ | 0.75 ± 0.25 |  | (2) |
| Intravenous (200 IU) inoculation | 22 | 10-55 | 8 (n = 2) or  15 (n = 20) | 2.08×10^5^ | 1.21 ± 0.12 |  | (2, 3) |
| Intravenous (500 IU) inoculation | 30 | 9-190 | 15 | 3.76×10^5^ | 1.46 ± 0.09 |  | (2) |
| Intravenous (2200 IU) inoculation | 4 | 192-387 | 11 (n = 2) or  12 (n = 2) | 9.97×10^4^ | 1.26 ± 0.10 |  | (2) |
| Stimulated primary cells (treated) | 3 | 1192-1305 | 2 | 1.42×10^3^ | 0.31 ± 0.01 | 0.82 ± 0.03 |  |
| SupT-R5 cell line (treated) | 3 | 2932-3023 | 2 | 8.69×10^2^ | 0.17 ± 0.002 | 0.99 ± 0.01 |  |
| Low-input sequencing | 10 | 29-42 |  | 5.37 | 0.013 ± 0.001 |  |  |
| Stimulated primary cells (untreated) | 3 | 664-772 | 4 | 1.55×10^3^ | 0.28 ± 0.01 | 0.50 ± 0.03 |  |
| SupT-R5 cell line (untreated) | 3 | 1614-1692 | 7 | 8.54×10^3^ | 0.45 ± 0.02 | 0.75 ± 0.02 |  |

*± standard error. ^Tail only removed from in vitro experiments.

1. Correlation between number of detected barcodes and inoculation size

Figure S1: Number of founding lineages is correlated with inoculation size. The number of unique barcodes detected within each intravenously inoculated animal plotted against the inoculation size used to infect the animal (reported in infection units (IU) as determined by TZM-bl infectivity assay). The Spearman correlation coefficient and corresponding p-value are listed.

1. Proof of log-normal distribution resulting from normally distributed delays

Here, we demonstrate that a normal distribution in time to the start of exponential growth of individual viral lineages generates a log-normal distribution in the size of viral lineages. To this end, first define $V_{0}$ as a set viral load such that for all larger viral loads, growth is exponential, and $t$ as the time post infection viral loads are measured. Also, let $V$ be the random variable for viral load of a lineage at time $t$, and $\tau$ be the normally distributed delay from infection to when that lineage has a viral load of $V_{0}$. Then, the duration said lineage has been growing exponentially is $t-\tau$, and

$$V=V_{0}e^{t-\tau}.$$

Therefore,

$$\ln\left( V \right)=\ln\left( V_{0} \right)+t-\tau.$$

Now, as it is assumed $\tau$ is normally distributed (i.e., $\tau\sim N\left( m,s^{2} \right)$),

$$\ln\left( V \right)\sim\ln\left( V_{0} \right)+t-N\left( m,s^{2} \right)=N\left( \ln\left( V_{0} \right)+t-m, s^{2} \right).$$

Hence, by definition, lineage sizes would be log-normally distributed.

1. Assay and viral variability insufficient to explain lineage size heterogeneity

In this supplement, we confirm that the observed lineage size heterogeneity is genuine and not the result of measurement error or a unique characteristic of the experimental model used. Specifically, we demonstrate that the two alternative hypotheses suggested in the main text; (i) measurement error due to PCR amplification bias and (ii) differences in viral fitness among barcoded clonotypes, are insufficient to explain the observed lineage size heterogeneity.

- 1. Assessment of the potential impact of PCR amplification bias

High variability in PCR amplification of lineages may have artificially increased the lineage size variance. If this was the case, then the size of individual lineages should not be correlated between samples taken from a single animal at different time points (since the sizes would be based on variability in the PCR amplification). We sequenced plasma virus from the 100 IU and two 200 IU intravenously inoculated animals six days after their first sequenced sample (day 8) and observed a strong correlation in lineage sizes between the two time points (Figure S1.1A; Pearson correlation coefficient of 0.95, p < 10^-24^) indicating observed lineage size heterogeneity is not solely due to variability in PCR amplification.

Figure S2.1: PCR amplification bias is insufficient to explain lineage size heterogeneity. (A) Positive Pearson correlation in the percentage of viral load composed of individual barcoded lineages between plasma samplings on days 8 and 14 in four intravenously inoculated animals. Two animals were inoculated with 100 IU (25428 and 25866) and two with 200 IU (26764 and 29445). Lineages newly detected on day 14 are indicated as below limit of detection (LOD) on day 8 and are not included in calculation of correlation coefficient and significance. (B) Empirical distributions in PCR amplification of single viral templates from 1:9.8 million dilutions of SIVmac239M2 barcoded viral stock.

In order to directly measure the variability in PCR amplification of individual viral templates we diluted the viral stock 9.8 million-fold, with the idea being that at this dilution we would obtain at most one template of a given barcode in a well. We considered 10 replicates and found that each replicate contained less than 0.03% of the stock barcodes (≤42 barcodes out of more than 140000 barcodes in the stock (2)), confirming that it was highly unlikely that any single barcode was present at more than one copy.

Following PCR amplification, the log_10_ sequence count for individual barcodes had an average variance of 0.013 ± 0.001 (log_10_ copies)^2^ (mean ± standard error) across the ten replicates (range 0.008 to 0.021; Figure S1.1B). This variability in amplification of individual templates is minor (1%) compared to the observed spread in lineage size in vivo, as illustrated in Fig 3A of the main text.

- 1. Assessment of replication bias among barcoded viral clonotypes

Another potential cause of lineage size heterogeneity not reported on in the Results of the main text is the potential variation in replicative capacity across viral lineages. If different clonotypes had different replicative fitness in this experimental model, faster replicating clonotypes would tend to be larger and grow to be even more dominant in the plasma viral load as infection progresses. Additionally, we would expect more fit barcodes to be detected in a larger proportion of animals and consistently be the dominant barcodes within those animals.

The same four animals that were sequenced at two separate time points and analysed in S1.1 can also be used to assess variation in barcode growth rate. Comparison of increases in barcoded clonotype viral loads from day 8 to 14 in these animals demonstrates that barcoded lineages in fact grow in parallel (Figure S1.2), suggesting that variation in replicative fitness is negligible.

Figure S2.2 Parallel growth of barcoded viral lineages in vivo. Viral load of individual barcoded lineages on 8 and 14 days post infection in four animals inoculated intravenously with 100 IU (25428 and 25866) or 200 IU (26764 or 29445). Most barcodes were detected at both time points, and lines connect viral loads on day 8 and 14 corresponding to the same barcode.

Furthermore, we saw no evidence of select barcodes being overrepresented across animals. In fact, only a single barcode was the largest clonotype in more than one animal (Figure S1.3A) and 71.5% of all barcodes detected in an intravenously inoculated animal were detected in only one animal (Figure S1.3B).

Figure S2.3 Different barcodes detected in different animals. Histograms for (A) the number of animals in which a barcode was the largest barcode or (B) simply detected in the plasma viral load. Histograms were generated based on data from all animals intravenously inoculated with SIVmac239M (n = 58).

Taken together, these data demonstrate that variation in viral fitness is not a driver of lineage size heterogeneity in this experimental model. However, in the context of exposure to a diverse viral pool, lineage size heterogeneity would most likely be exacerbated by discrepancies in replicative fitness.

1. Lineage size distribution following untreated in vitro replication

In this Supplement, we provide evidence that accumulated lineage size heterogeneity resulting from multiple rounds of replication is insufficient to explain the level of heterogeneity observed in vivo. That is, given a distribution in viral production by individual cells, lineage size heterogeneity increases over the course of the initial rounds of replication. However, when in vitro infected cells were allowed to proceed through multiple rounds of infection (ie: not treated to block infection), the observed final lineage size heterogeneity for primary cells and the SupT1-R5 cell line remained much narrower than was observed in vivo (S2.1). Additionally, a simulation of lineage size heterogeneity over multiple rounds of replication also indicates that in order to achieve the level of heterogeneity observed in vivo, the variance in viral production by individual cells must be wider than what we observed in primary cells and the SupT1-R5 cell line (S2.2).

- 1. Observed lineage size heterogeneity following ongoing in vitro infection

The heterogeneity in lineage size observed following multiple rounds of replication is illustrated in Figure S2.1.

Figure S3.1: Lineage size heterogeneity following untreated viral replication in vitro. Stimulated primary cells (A) or SupT1-R5 cells (B) were infected with SIVmac239V67M. Inoculum was washed out after ~18 hours, and supernatant was subsequently washed at 24 hours post infection and 24-hourly thereafter. Lineage sizes were measured via Illumina sequencing of the supernatant RNA at 4 days and 7 days after infection in the stimulated primary cells and SupT1-R5 cells, respectively. Empirical distributions (faded lines) and best fit distributions (dark lines) of lineage size (log_10_-scale) in each of the three stimulated primary cell (A, red lines), and SupT1-R5 (B, grey lines) wells are plotted. Main figures focus on the log-normal component of the distributions, while insets display the full data sets and fits (including the tail).

Here we explain how we estimated the potential contribution of multiple rounds of viral replication to the overall dissemination bottleneck. This estimate is based on comparison of lineage size diversity on day two of anti-retroviral treated in vitro wells (i.e., viral production by single cells; Fig 2 of the main text) to that on day four or seven of untreated wells (i.e., after multiple rounds of viral replication has occurred; Figure S2.1). Variance in log_10_ lineage size ($\sigma_{10}^{2}$) increased from single cell production to following multiple rounds of replication in SupT1-R5 cells but decreased in the stimulated primary cell experiments. Therefore, we use the increase in $\sigma_{10}^{2}$in the SupT1-R5 cell data to estimate an upper limit of the contribution of multiple rounds of viral replication to the overall dissemination bottleneck. Specifically, the difference in $\sigma_{10}^{2}$ in treated and untreated experiments gives an estimate of the $\sigma_{10}^{2}$ caused by multiple rounds of stochastic viral replication, and the ratio of this difference and the $\sigma_{10}^{2}$of the intravenously inoculated animals gives the estimated proportional contribution of multiple rounds of replication to the overall dissemination bottleneck.

We first removed bias due to inherent variability in PCR amplification by background subtracting the variance in log_10_ lineage size in the PCR amplification data ($\sigma_{10;PCR}^{2}$ estimated from the low-input sequencing; S1 Text) from the mean observed $\sigma_{10}^{2}$ for each experimental set up (similar to comparisons in the main text). For the purpose of simplicity for the remainder of this Supplement, we will refer to this background subtracted variance in log_10_ lineage sizesimply as $\hat{\sigma}_{10,j}^{2}$ for experimental set up $j$, where

$$\hat{\sigma}_{10,j}^{2}= \sigma_{10,j}^{2}-\sigma_{10,PCR}^{2}.$$

We then estimated the upper limit on the proportional contribution of multiple rounds of replication to the overall dissemination bottleneck by considering the ratio of the increase in $\sigma_{10}^{2}$ from treated to untreated in vitro cell line experiments to the $\sigma_{10}^{2}$ of the intravenously inoculated animals, i.e.

$$Estimated contribution of multiple rounds of replication=\frac{\hat{\sigma}_{10,CLU}^{2}-\hat{\sigma}_{10,CL}^{2}}{\hat{\sigma}_{10,IV}^{2}},$$

where $\hat{\sigma}_{10,CLU}^{2}, \hat{\sigma}_{10,CL}^{2}$, and $\hat{\sigma}_{10,IV}^{2}$ are the (background subtracted) variances in log_10_ lineage size measured in the untreated cell line in vitro wells, treated cell line in vitro wells, and the intravenously inoculated animals, respectively.

- 1. Simulated accumulation of lineage size heterogeneity

Here, we use a simulation to assess how much lineage size heterogeneity increases over the course of multiple rounds of viral replication, and if this accumulated heterogeneity is sufficient to reach the heterogeneity observed in vivo. Figure S2.2 suggests that in the absence of any other factors (such as differences between anatomical sites) a much larger variance in log_10_ viral production than we observe for production by individual cells would be required in order for the final heterogeneity to match that observed in vivo.

Figure S3.2: Simulated final lineage size heterogeneity following multiple rounds of replication. Viral replication was simulated using an average basic reproductive number of 8 (median value estimated by Ribeiro et al. (4); solid line), 4 (dashed line), and 16 (long dashed line). The observed variance in log_10_ lineage size ($\sigma_{10}^{2}$) following untreated replication in primary cells (orange) and the SupT1-R5 (purple) from S2.1 is plotted vs. the corresponding observed variance in viral production by single cells (standard errors are indicated by error bars). The blue line indicates the average $\sigma_{10}^{2}$ observed in I.V. inoculated animals (1.24 ± 0.54 (log_10_ copies/ml)^2^).

The simulation results presented in Figure S2.2 were generated in the following manner. A simulation was run for each combination of $R_{0}$ and initial variance in log_10_ lineage size ($\sigma_{10,1}^{2}$), and for each simulation, 10^4^ lineages were established. To explain the simulation of each lineage, define the random variables $C_{k}$ and $V_{k}$ be the number of infected cells and “virus” produced at generation $k$, and let $c_{k}$ and $v_{k}$ represent the corresponding “observed” values. Following the first generation, where each lineage was established with a single infected cell ($c_{1}=1$), for each generation, the number of cells to be infected was assumed to come from a Poisson distribution with mean ($\lambda$) dependent on the number of viruses produced in the previous generation

$$C_{k}\sim Poisson\left( \lambda=\beta V_{k-1} \right),$$

where $\beta$ is the number of cells infected per virion. The total “viral production” $V_{k}$ is then the sum of the production from each cell. To calculate $V_{k}$, let $V_{k,l}$ be the viral production by the $l^{th}$ cell in the $k^{th}$ generation,

$$V_{k}=\sum_{l=1}^{c_{k}} V_{k,l}.$$

Based on our in vitro experiments, we assume, $V_{k, l}$ is lognormally distributed

$$V_{k,l}\sim LN\left( \mu=0,\sigma^{2}=\left( \ln\left( {10}^{\sigma_{10,1}} \right) \right)^{2} \right).$$

We arbitrarily set the mean logarithmic value ($\mu$) to 0, as inversely varying $\mu$ and $\beta$ provide the same expected number of infected cells ($\lambda$). Hence, the exact value of $\mu$ is irrelevant. As the following computation is simpler using the lognormal distribution parameters on the natural log scale, we convert from variance on the log_10_ scale (used in the rest of the manuscript) to variance on the natural log scale, $\sigma^{2}=\left( \ln\left( {10}^{\sigma_{10,1}} \right) \right)^{2}.$

To set $\beta$, we use the definition of the basic reproduction number ($R_{0}$). That is the number of infected cells is expected to increase $R_{0}$ fold with each generation

$$E\left( C_{k} \right)=R_{0}c_{k-1}.$$

Then, based on the relation between $C_{k}$ and $V_{k}$,

$$E\left( C_{k} \right)=E\left( \beta V_{k-1} \right)=\beta E\left( \sum_{l=1}^{c_{k-1}} V_{k,l} \right)=\beta\sum_{l=1}^{c_{k-1}} E\left( V_{k,l} \right)=\beta c_{k-1}E\left( V_{k,l} \right)$$

$$=\beta c_{k-1}e^{\sigma^{2}/2}$$

where the last equality results from the fact that the mean of the lognormal distribution $LN\left( \mu,\sigma^{2} \right)$ is $e^{\mu+\frac{\sigma^{2}}{2}}$. Thus,

$$R_{0}c_{k-1}=\beta e^{\sigma^{2}/2}c_{k-1}$$

or

$$\beta=R_{0}e^{-\sigma^{2}/2}.$$

For each combination of $R_{0}$ and single cell production variance ($\sigma_{10}^{2}$), the number of produced viruses and infected cells of each lineage was simulated through multiple generations until $\sigma_{10}^{2}$ plateaued (6, 8, and 9 generations for $R_{0}$s of 4, 8, and 16, respectively).

References

1. Deleage C, Immonen TT, Fennessey CM, Reynaldi A, Reid C, Newman L, Lipkey L, Schlub TE, Camus C, O'Brien S, Smedley J, Conway JM, Del Prete GQ, Davenport MP, Lifson JD, Estes JD, Keele BF. 2019. Defining early SIV replication and dissemination dynamics following vaginal transmission. Sci Adv 5:eaav7116.

2. Khanal S, Fennessey CM, O'Brien SP, Thorpe A, Reid C, Immonen TT, Smith R, Bess JW, Jr., Swanstrom AE, Del Prete GQ, Davenport MP, Okoye AA, Picker LJ, Lifson JD, Keele BF. 2019. In vivo validation of the viral barcoding of SIVmac239 and the development of new barcoded SIV and subtype B and C SHIVs. J Virol doi:10.1128/JVI.01420-19.

3. Okoye AA, Duell DD, Fukazawa Y, Varco-Merth B, Marenco A, Behrens H, Chaunzwa M, Selseth AN, Gilbride RM, Shao J, Edlefsen PT, Geleziunas R, Pinkevych M, Davenport MP, Busman-Sahay K, Nekorchuk M, Park H, Smedley J, Axthelm MK, Estes JD, Hansen SG, Keele BF, Lifson JD, Picker LJ. 2021. CD8+ T cells fail to limit SIV reactivation following ART withdrawal until after viral amplification. J Clin Invest 131.

4. Ribeiro RM, Qin L, Chavez LL, Li D, Self SG, Perelson AS. 2010. Estimation of the initial viral growth rate and basic reproductive number during acute HIV-1 infection. J Virol 84:6096–102.
